# Quantification of tremor using consumer product accelerometry is feasible in patients with essential tremor and Parkinson’s disease: a comparative study

**DOI:** 10.1186/s40734-020-00086-7

**Published:** 2020-04-07

**Authors:** Emilie M. J. van Brummelen, Dimitrios Ziagkos, Wadim M. I. de Boon, Ellen P. Hart, Robert J. Doll, Teppo Huttunen, Petteri Kolehmainen, Geert Jan Groeneveld

**Affiliations:** 1 Centre for Human Drug Research, Zernikedreef 8, Leiden, 2333 CL The Netherlands; 2 4Pharma Ltd, Turku, Finland; 3 Make Helsinki Oy, Helsinki, Finland; 4 Department of Anesthesiology, Leiden University Medical Center (LUMC), Leiden, The Netherlands

**Keywords:** Tremor, Validation, App, Wearable, Parkinson’s disease, Accelerometer, Essential tremor, Tremography

## Abstract

**Background:**

To quantify pharmacological effects on tremor in patients with essential tremor (ET) or Parkinson’s Disease (PD), laboratory-grade accelerometers have previously been used. Over the last years, consumer products such as smartphones and smartwatches have been increasingly applied to measure tremor in an easy way. However, it is unknown how the technical performance of these consumer product accelerometers (CPAs) compares to laboratory-grade accelerometers (LGA). This study was performed to compare the technical performance of CPAs with LGA to measure tremor in patients with Parkinson’s Disease (PD) and essential tremor (ET).

**Methods:**

In ten patients with PD and ten with ET, tremor peak frequency and corresponding amplitude were measured with 7 different CPAs (Apple iPhone 7, Apple iPod Touch 5, Apple watch 2, Huawei Nexus 6P, Huawei watch, mbientlabMetaWear (MW) watch, mbientlab MW clip) and compared to a LGA (Biometrics ACL300) in resting and extended arm position.

**Results:**

Both in PD and ET patients, the peak frequency of CPAs did not significantly differ from the LGA in terms of limits of agreement. For the amplitude at peak frequency, only the iPhone and MW watch performed comparable to the LGA in ET patients, while in PD patients all methods performed comparable except for the iPod Touch and Huawei Nexus. Amplitude was higher when measured with distally-located CPAs (Clip, iPhone, iPod) compared with proximally-located CPAs (all watches). The variability between subjects was higher than within subjects for frequency (25.1% vs. 13.4%) and amplitude measurement (331% vs. 53.6%). Resting arm position resulted in lower intra-individual variability for frequency and amplitude (13.4 and 53.5%) compared to extended arm position (17.8 and 58.1%).

**Conclusions:**

Peak frequencies of tremor could be measured with all tested CPAs, with similar performance as LGA. The amplitude measurements appeared to be driven by anatomical location of the device and can therefore not be compared. Our results show that the tested consumer products can be used for tremography, allowing at-home measurements, in particular in studies with a cross-over or intra-individual comparison design using the resting arm position.

**Trial registration:**

This trial was registered in the Dutch Competent Authority (CCMO) database with number NL60672.058.17 on May 30th 2017.

## Introduction

A tremor is a rhythmic involuntary oscillatory movement that can affect body parts such as hands, arms, eyes and legs [12]. Most often, tremor is classified as a symptom of essential tremor (ET) or Parkinson’s Disease (PD). Other classifications of tremor include psychogenic tremor, dystonic tremor, orthostatic tremor or tremor as a symptom of multiple sclerosis [17, 19].

Quantification of tremor is essential to determine the severity of tremor and to evaluate responses to treatment. Objective quantification of tremor can be done with accelerometers, which measure dynamic and static accelerations. These measurements are generally done for both resting and action tremors separately, and provide frequency and amplitude of the tremor [1, 21]. While the frequency can be used to discriminate between different types of tremor, the amplitude is mainly used to classify disease and to follow disease progression [10, 11].

In various clinical studies, laboratory-grade accelerometers (LGAs) have been successfully used to assess tremor and to quantify treatment effects [3, 5, 16, 22]. Advances in technology have led to integration of accelerometers in consumer products (consumer product accelerometers, CPAs) such as smartphones and smartwatches [8]. Integrated CPAs allow for easy-to-use domestic measurements, independent of clinical facilities, and may therefore provide a cost-effective alternative for LGA measurements [14–16].

Several studies have been performed with mobile applications to measure tremor in patients with ET or PD. Fraiwad et al. reported high accuracy (95%) of the Samsung Galaxy SII with the Android Mobile Sensor UDP application [8]. Wile et al. found that tremography with a smartwatch (WIMM One) had nearly perfect concordance with a LGA [20]. Additionally, Joundi et al. showed that accelerometry with iPhone closely matched electromyogram measurements [13]. However, a comparison with a non-consumer grade accelerometer has not been reported before. Hence, it remains unknown how the technical performance of the CPAs compares to LGAs. Therefore, this exploratory study was initiated to provide a thorough comparison of technical feasibility between eight different CPAs and a LGA of tremor measurement in patients with ET or PD.

## Materials and methods

### Subjects

Patients were eligible if they were 18 to 80 years of age (inclusive), had a diagnosis of classic ET or a clinical diagnosis of PD with Hoehn and Yahr stage ≤ III [9], and had tremor in at least one hand, regardless of current therapy. Any other active or chronic disease was not allowed. All patients provided written informed consent before any study procedure was carried out. The trial was conducted in accordance with Good Clinical Practice and the protocol was approved by the ethics committee of the Leiden University Medical Centre.

### Measurements

**Fig. 1:**
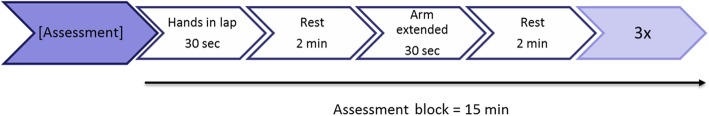
Sequence and timeline of one assessment block

**Fig. 2:**
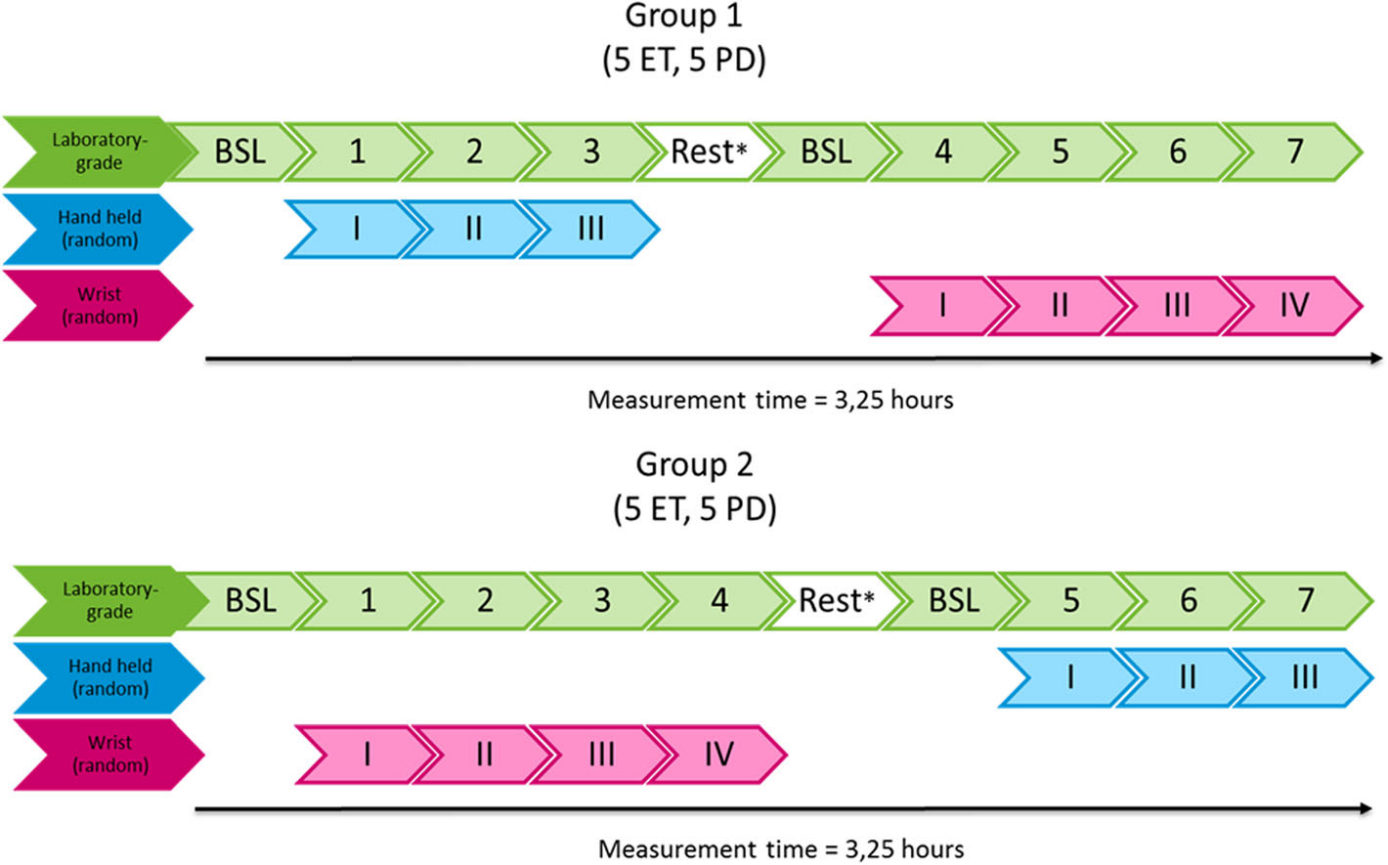
Timeline overview of all groups containing all assessments. ^*^A break of a minimum of 30 min up to 1 h was allowed if needed. BSL = Baseline/reference laboratory grade accelerometer, ET = Essential Tremor, PD = Parkinson’s Disease

In all patients, simultaneous tremor assessments were performed with the LGA (Biometrics ACL300, 3-axes accelerometer), using the DataLINK data logger for data capture, and seven different CPAs (Apple iPhone 7, Apple iPod Touch 5, Apple Watch 2, Huawei Nexus 6P, Huawei watch, mbientlab MetaWear watch, and mbientlab MetaWear clip). For the CPAs, data were captured using a Make Helsinki app or a Centre for Human Drug Research app (for the iPod Touch 5 only). Both LGAs and CPAs measured tri-axial acceleration and were used simultaneously. The LGA was attached to the dorsal side of the hand that was most affected by tremor (or the dominant hand in case of equal tremor at both sides), in line with the third digit. CPAs were held in a natural position (supination, without flexion/extension of the hand, with the third digit in line with the lower arm) in the hand (phones), attached to the wrist (watches), or clipped to the distal phalanx of the third digit (mbient MW clip) of the same arm as the LGA. Two standardized measurements were performed for 30 s, one in resting arm position and one in extended arm position (supplemental Figures 7, 8, 9, 10 and 11). It should be noted that although the term ‘resting arm position’ is used, the position supports the forearm but still requires some activation of the wrist and forearm. Each assessment was performed three times with 2 min of rest in between (Fig. 1). The patients were randomized into two equal subgroups. The first group started assessments with baseline measurements with the LGA only and continued with both the LGA and the hand-held products. The order of CPAs was randomized. Once the hand-held products were assessed, patients had a break of 30 min up to 1 h. Thereafter, they had a new baseline measurement with the LGA only and continued with both LGA and the wrist- or finger worn products in randomized order. The second group first started with baseline measurements with the LGA, continued with both LGA and wrist- or finger worn products and finished with LGA and the hand-held products (Fig. 2). The first assessment was defined as t = 0 h. The baseline assessments were defined as all assessments executed with the LGA.

### Endpoints and analysis

The primary endpoint was the peak frequency (Hz). The secondary endpoint was the amplitude (mg^2^/Hz) at the peak frequency. The endpoints were calculated using MATLAB 9.1 (MathWorks, Inc., Natick, MA). For devices with a variable sampling frequency (i.e., Apple products and the MW devices, see Table 1), samples were linearly interpolated prior to further analysis. Each axis was bandpass filtered between 1 and 20 Hz using a zero-phase third order Butterworth filter. After calculating the Power Spectrum Density for each axis, the L1 norm was calculated effectively combining the three axis. The peak frequency value is defined as the frequency at the maximum value of the L1-PSD. The amplitude at the peak frequency is defined as the peak amplitude.

**Table 1:** Sample frequency, standard deviation (SD), weight and range per accelerometer. ^a^Range as defined in specifications, exact setting could not be found

Consumer product	Data acquisition software	Number of measurements (n)	Sample frequency (Hz)	SD (Hz)	Weight device (g)	Range (±G)
Standard	Biometrics	840	1000	0.0	10	10
iPhone	Make Helsinki	120	329	41.7	138	2–16^a^
iPod	CHDR	120	98	31.7	88	2–16^a^
Apple Watch	Make Helsinki	120	52	5.8	28	2–16^a^
Huawei Nexus	Make Helsinki	120	20	0.0	178	2–16^a^
Huawei watch	Make Helsinki	119	20	0.0	60	2–16^a^
MW watch	Make Helsinki	118	103	23.3	17	2
MW clip	Make Helsinki	120	101	0.4	11	2

Forest plots describing the 95% limits of agreement were used to compare the technical performance of the accelerometers. A mixed model analysis of covariance was used to establish whether significant patient group effects could be detected. A linear mixed model was fit that takes into account the repeated measurements within each position. The method has been described in detail earlier [2, 4]. Briefly, this method calculates the limits of agreement using the formula: ${\hat{\alpha}}_1-{\hat{\alpha}}_2\pm \sqrt{2\ast {\hat{\tau}}^2+{\hat{\sigma_1}}^2+{\hat{\sigma_2}}^2}$, where *τ*, *σ*_1_, *σ*_2_ are the variance components calculated with the mixed model reflecting the consumer product by subject interaction variance, the residual LGA variance, and the residual CPA variance respectively. All statistical programming was conducted with SAS 9.4 for Windows (SAS Institute Inc., Cary, NC, USA). The mixed model analysis was performed using proc. mixed (SAS Institute Inc., Cary, NC, USA). The variability of measurements within subjects (intra-individual variability) was calculated, based on three measurements with each device and expressed as coefficient of variation. The minimum detectable effect size is calculated using the covariance parameter estimates from the mixed model output capturing the between and within subject variance times the square root of 1/N where N is the number of subjects. This is then multiplied by the sum of two t-distributions at alpha (significance level of 0.05) and at 2*(1-P) where P is the power of 0.80 with N-1 degrees of freedom.

## Results

Ten patients with ET (3 male, 7 female) and ten with PD (8 male, 2 female) were included. Mean age was 44.5 (±17, range 23–78) years for ET and 63.2 years for PD (±11, range 43–78). All measurements were performed as planned. Mean sample frequencies and standard deviations per device are described in Table 1.

### Peak frequency and amplitude at peak frequency compared to LGA

**Fig. 3:**
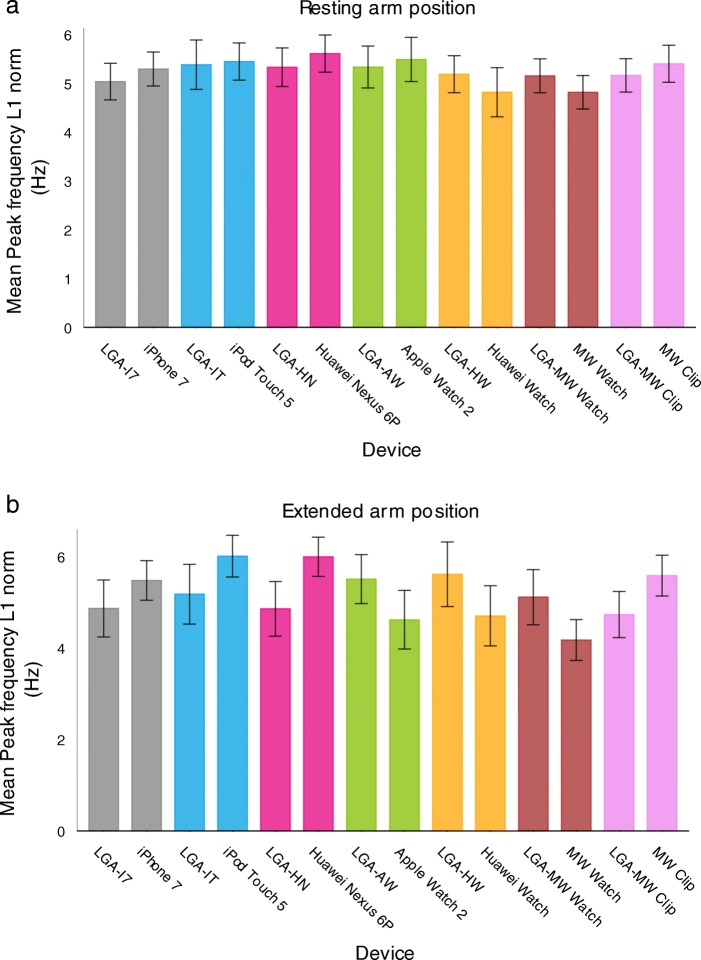
Mean peak frequency of consumer product accelerometers versus laboratory grade accelerometer in patients with Essential Tremor and Parkinson’s Disease in resting arm position (**a**) and extended arm position (**b**). AW = Apple watch 2; HN = Huawei Nexus 6P; HW = Huawei watch; IT = iPod Touch 5; I7 = iPhone 7; MW Clip = Meta Wear Clip; MW Watch = Meta Wear watch. Every measurement was combined with the LGA to allow simultaneous measurements. The peak frequency measured with the LGA showed slight changes (increases and decreases) over time

**Fig. 4:**
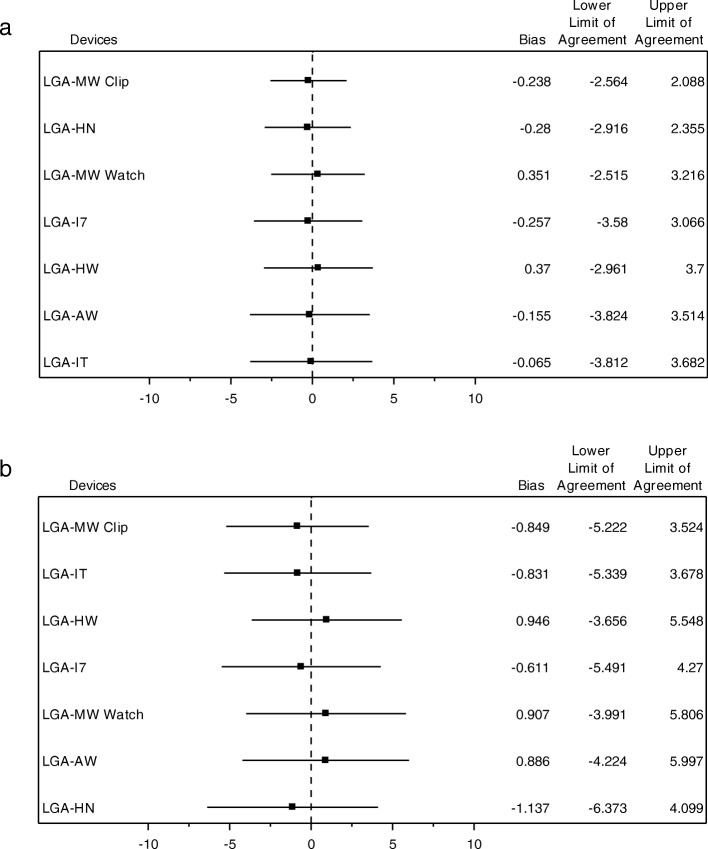
**a** Limits of agreement of peak frequency between consumer product accelerometers and the laboratory grade accelerometer in patients with Essential Tremor and Parkinson’s Disease in resting arm position. AW = Apple watch 2; HN = Huawei Nexus 6P; HW = Huawei watch; IT = iPod Touch 5; I7 = iPhone 7; MW Clip = Meta Wear Clip; MW Watch = Meta Wear watch. **b** Limits of agreement of peak frequency between consumer product accelerometers and the laboratory grade accelerometer in patients with Essential Tremor and Parkinson’s Disease in extended arm position. AW = Apple watch 2; HN = Huawei Nexus 6P; HW = Huawei watch; IT = iPod Touch 5; I7 = iPhone 7; MW Clip = Meta Wear Clip; MW Watch = Meta Wear watch

In ET patients, peak frequency ranged between 4.4 and 6.6 Hz and in PD patients between 3.9 and 5.5 Hz. Peak frequency (Fig. 3a and b) including the limits of agreement with any consumer product did not significantly differ from the LGA in resting arm position (Fig. 4a) nor in extended arm position (Fig. 4b).

**Fig. 5:**
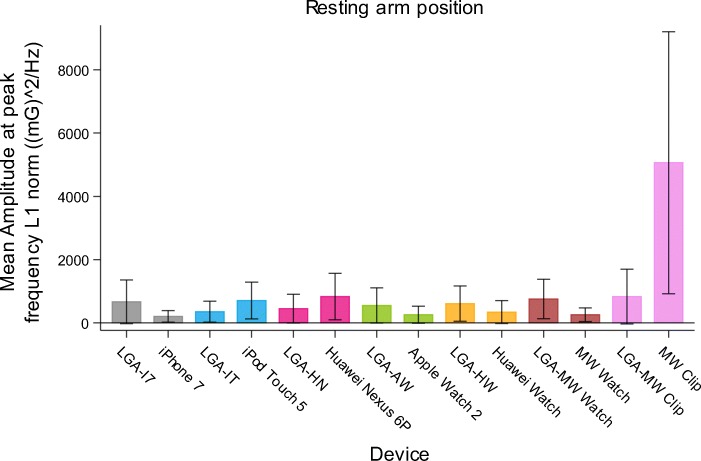
Amplitude at peak frequency in all patients in resting arm position for each pair of reference measurements with the laboratory grade accelerometer and consumer product accelerometer

**Fig. 6:**
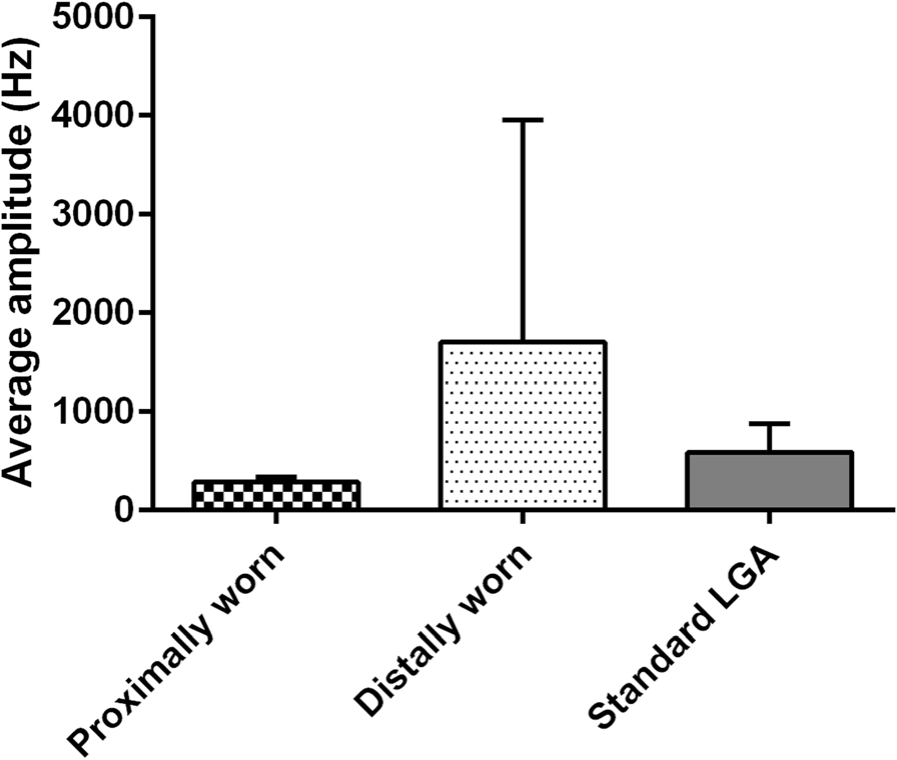
Amplitude at peak frequency in all patients in resting arm position for proximally-worn accelerometers (Apple Watch, Huawei Watch and MW Watch) compared with distally-worn accelerometers (MWClip, iPod, iPhone and Huawei Nexus) and the standard laboratory grade accelerometer. Amplitude at peak frequency tended to be higher after measurement with distally-worn accelerometers compared to proximally-worn accelerometers

Amplitude at peak frequency tended to be higher after measurement with distally-located accelerometers (range 204.9–5061) being the MW Clip, iPod, iPhone and Huawei Nexus compared to proximally-located accelerometers (range 254.0–341.0) being the Apple Watch, Huawei Watch and MW Watch (Figs. 5 and 6). For both the ET and PD patients, a similar tendency is seen when the amplitude at peak frequency values are compared to LGA. In the distally-located devices the values are notably higher than the LGA values (mean differences are negative), and somewhat more pronounced in the extended arm position. In the proximally-located devices the amplitude at peak frequency values are more stable irrespective of the arm position and somewhat lower as compared to LGA values (supplemental Figures 1, 2, 3, 4, 5 and 6 providing the limits of agreement for the log-transformed values).

### Variability of peak frequency and amplitude measurements

Overall, the intra-individual variability of both frequency and amplitude measurements was lower in resting arm position (13.4 and 53.5% respectively) than in extended arm position (17.8 and 58.1%).

The intra-individual variability of peak frequency was on average 13.4% (range 8.79–23.3%) in resting arm position (Table 2), and 17.8% (range 10.9–25.4%) in extended arm position (Table 3). The lowest variability based on both extended and resting arm position was observed with the iPhone 7. The inter-individual variability was on average 25.1% (range 20.7–32.3%) in resting arm and 33.0% (range 21.6–49.7%) in extended arm position. The MW clip showed the lowest inter-individual variability based on both positions. For LGA, the intra-individual variability for frequency measurements was on average 16.3 and 21.7% in resting and extended arm position respectively, compared to 12.6 and 16.7%, for all CPAs. The minimum detectable change in frequency ranged from 0.7 Hz in resting arm position with the MW watch to 1.5 Hz with the Apple watch in extended arm position (supplementary Table 1).

**Table 2:** Mean peak frequency L1 norm measured with each consumer product accelerometer (CPA) and laboratory grade accelerometer (LGA) as reference in resting arm position based on 20 patients

Location	Device	Mean Peak Frequency (Hz)	Intra-individual variability Coefficient of variation (%)	Inter-individual variability Coefficient of variation (%)
Distal	Reference LGA^a^	4.76	23.28	20.72
Distal	Reference LGA^b^	5.45	9.23	29.52
Distal	iPhone 7	5.29	8.66	23.19
Distal	iPod Touch 5	5.44	10.77	24.32
Distal	Huawei Nexus 6P	5.6	8.79	24.89
Distal	MW Clip	5.4	10.98	24.18
Proximal	Apple Watch 2	5.48	14.93	25.36
Proximal	Huawei Watch	4.81	18.45	32.27
Proximal	MW Watch	4.79	15.30	21.61

^a^Reference LGA measurement prior to CPA hand-held devices ^b^Reference LGA measurement prior to CPA wrist-worn devices

**Table 3:** Mean peak frequency L1 norm measured with each consumer product accelerometer (CPA) and laboratory grade accelerometer (LGA) as reference in extended arm position based on 20 patients

Location	Device	Mean Peak Frequency (Hz)	Intra-individual variability Coefficient of variation (%)	Inter-individual variability Coefficient of variation (%)
Distal	Reference LGA^a^	5.61	23.25	33.19
Distal	Reference LGA^b^	5.35	20.16	36.40
Distal	iPhone 7	5.48	10.95	28.07
Distal	iPod Touch 5	6.01	10.46	26.16
Distal	Huawei Nexus 6P	5.99	15.64	22.13
Distal	MW Clip	5.58	17.93	21.57
Proximal	Apple Watch 2	4.62	17.68	49.74
Proximal	Huawei Watch	4.68	25.42	44.09
Proximal	MW Watch	4.23	19.10	35.96

^a^Reference LGA measurement prior to CPA hand-held devices ^b^Reference LGA measurement prior to CPA wrist-worn devices

The measurement of amplitude at peak frequency had a high variability relative to the absolute amplitude in all patients with all devices. Intra-individual variability was on average 53.6% (range 26.6–89.5%) in resting arm (Table 4) and 58.1 (range (27.9–124%) in extended arm position (Table 5). The inter-individual variability was on average 331% (range 293–383%) in resting arm and 373% (range 316–431%) in extended arm position. The iPhone 7 showed the lowest intra-individual variability and the LGA the lowest inter-individual variability for amplitude measurements. For LGA, the intra-individual variability for amplitude measurements was on average 88,8 and 32.6% in resting and extended arm position respectively, compared to 43.6 and 65.4% for all CPAs. The minimum detectable change in amplitude ranged from 42 (mG^2)/Hz in extended arm position with the LGA to 10,247 (mG^2)/Hz with the MW clip in resting arm position (supplementary Table 1).

**Table 4:** Mean amplitude at peak frequency measured with each consumer product accelerometer (CPA) and laboratory grade accelerometer (LGA) as reference in resting arm position based on 20 patients

Location	Device	Mean (mG^2/Hz)	Intra-individual variability Coefficient of variation (%)	Inter-individual variability Coefficient of variation (%)
Distal	Reference LGA^a^	794.58	88.17	293.11
Distal	Reference LGA^b^	384.21	89.47	342.36
Distal	iPhone 7	204.92	26.78	345.72
Distal	iPod Touch 5	706.81	45.08	311.90
Distal	Huawei Nexus 6P	832.41	54.85	307.80
Distal	MW Clip	5061.86	41.69	306.48
Proximal	Apple Watch 2	257.64	57.28	370.40
Proximal	Huawei Watch	341.01	52.58	383.31
Proximal	MW Watch	253.96	26.62	325.83

^a^Reference LGA measurement prior to CPA hand-held devices ^b^Reference LGA measurement prior to CPA wrist-worn devices

**Table 5:** Mean amplitude at peak frequency measured with each consumer product accelerometer (CPA) and laboratory grade accelerometer (LGA) as reference in extended arm position based on 20 measurements

Location	Device	Mean (mG^2/Hz)	Intra-individual variability Coefficient of variation (%)	Inter-individual Coefficient of variation (%)
Distal	Reference LGA^a^	38.27	27.91	322.91
Distal	Reference LGA ^b^	20.31	37.26	316.00
Distal	iPhone 7	149.77	44.39	328.17
Distal	iPod Touch 5	869.93	51.00	403.96
Distal	Huawei Nexus 6P	666.42	66.79	407.66
Distal	MW Clip	1656.51	90.70	400.59
Proximal	Apple Watch 2	31.62	42.57	376.02
Proximal	Huawei Watch	67.34	124.33	431.74
Proximal	MW Watch	34.24	37.74	374.98

^a^Reference LGA measurement prior to CPA hand-held devices ^b^Reference LGA measurement prior to CPA wrist-worn devices

## Discussion

In this study, the peak frequency and the corresponding amplitude measurements performed with CPAs were compared to those performed with a LGA.

The peak frequencies were slightly higher for patients with ET (4.4–6.6 Hz) compared to those of patients with PD (3.9–5.5) which is in line with previously reported values [7, 18]. In both patient groups, we found that all consumer products provided similar results as the LGA in terms of peak frequency in both resting and extended arm positions. This indicates that the use of consumer products may be technically feasible to measure peak frequency of tremor in ET and PD patients.

The secondary endpoint in this study was amplitude at peak frequency. This amplitude suggests to be driven by the anatomical location where the sensor is worn (Fig. 6). The more distally worn sensors (MW Clip, iPod, iPhone, Nexus) tend to measure a higher amplitude, compared to more proximally worn sensors (Apple watch 2, Huawei watch, MW watch). This may explain why the Nexus, iPod, iPhone and MW clip have significantly different results in ET patients compared to the LGA, which is placed on dorsal side of the hand. This is expected since the detected amplitude is dependent on the distance from the axis of rotation [6].

Apart from comparing the performance of CPAs with LGA, the results of this study give insight the variability of each device in different positions (resting arm and extended arm). This information can be used to determine how the CPAs can be optimally used, in e.g. clinical trials or at-home measurements. Firstly, we found that for all devices the inter-subject variability was high (up to 33 and 44% for resting and extended arm position for frequency and up to 383 and 431% for resting and extended arm position amplitude measurements) while intra-subject variability was much lower although still relatively high (up to 23.3 and 25.4% for resting and extended arm position frequency measurements and 89.5 and 124.3% for amplitude measurements). Hence, results between individuals are difficult to compare, also in view of different types of tremor between subjects, while intra-individual comparisons are more accurate. Based on these results, for tremor measurements in clinical (pharmacological) studies, a cross-over setting or intra-individual comparison would be most feasible when using CPAs. Such designs will allow a minimum number of subjects needed to demonstrate changes in tremor, or effects on tremor of anti-tremorogenic drugs. The resting arm position seems better than the extended arm position for tremor measurements because the variability is lower of both peak frequency and amplitude at peak frequency measurements. A low variability will allow better detection of drug-effects on tremor amplitude with a limited number of subjects. Remarkably, the intra-individual variability of the CPAs tend to be lower than that of the LGA, except for amplitude measurements in the extended arm position. This indicates that the use of CPAs can lead to less variable measurements than with LGA, supporting again the use of the resting arm position. Amplitude measurements in extended arm position may be more variable with CPAs than the LGA, although frequency measurements are still comparable to LGA and less variable than LGA.

The high variability of the measurements, in particular the amplitude measurements, do warrant cautious interpretations of the data. When using amplitude measurements in clinical studies, a cross-over setting is needed to reduce the variability. However, the power to detect small –maybe not clinically meaningful- changes in amplitude will be relatively low. Based on the calculated minimum detectable change in amplitude, the LGA is the most sensitive to change (42 (mG^2)/Hz) while the Apple Watch is the most sensitive from the CPAs (78(mG^2)/Hz. A recommendation regarding which device would be optimal is not made in this manuscript. Each of them can provide a peak frequency measurement similar to the LGA. However, each of them has a slightly different profile with regards to variability and amplitude measurements. Depending on the objectives of the measurements and the patient preference, the most suitable device can be chosen. To detect a change in frequency, the MW watch would be most sensitive (minimum detectable change 0.7).

A weakness of this study is that all data are obtained in a very controlled, clinical setting with clear instructions and monitoring of the patients. In real-life and in future clinical studies, CPAs may be used in an at-home setting. While our results indicate that the tested CPAs are feasible for tremor measurements, it should be noted that at-home measurements may give rise to more variability and potentially different results. Clear instructions for patients on how to use the device and how to maintain standardized positions are needed. Additionally, the accelerometers used in this trial have some technical drawbacks. Firstly, the sampling frequency of some devices, in particular the Huawei Nexus and Huawei Watch, was low (20 Hz). Although for the iPhone and iPod, sampling frequency was higher, they demonstrated large sampling variability. This may have increased the inaccuracy of the measurements corresponding to these devices in this study. Taking into account that multiple measurements were done per subject, we expect that this inaccuracy is reflected in the data and the intra-individual comparisons are expected to provide representative results of these methods. Secondly, as tremor movements contain rotational movements, a gyroscope provides added value for quantification. In addition, the impact of gravity on the accelerometers could not be avoided but does not hinder the intra-individual comparisons we made. Therefore, gyroscopes are of added value to tremor quantifications, but accelerometers demonstrate to be useful for the comparison to the LGA, which was the purpose of this study.

We aimed to include a population that is representative for the real-life setting. This resulted in a group of men and women within the age range of 23–78 years. The heterogeneity of this population may have had an impact on the absolute tremor frequencies that we measured. However, we do not expect that this has had an impact on the comparison in performance between the LGA and CPAs because we have performed intra-individual comparisons. This intra-individual comparison makes the outcome insensitive to differences between subjects such as age, sex, and treatment. In a future study, a correlation with clinical scores may be provided such as the TETRAS score to provide a more in-depth profile of the CPA measurements. This was however beyond the scope of this study, in which we focused on the technical feasibility of measurements with CPAs.

Furthermore, it should be noted that the sample size of this study was limited due to the exploratory set-up of this study. However, still a large dataset was obtained that could be used for intra-individual comparisons. This is because tremor was measured in triplicate with each of the devices in two positions, in each subject. This yielded 840 measurements with the LGA, and about 120 measurements with each CPA. Each measurement with CPAs was performed simultaneously with the LGA device, which was regarded the reference measurement. The peak frequency measured with the LGA showed slight changes (increase and decreases) over time (Fig. 3). This could be an effect of tremor change over time during the course of the experiment. We expect that the change over time of the baseline measurement does not have an impact on our findings, because the CPA measurements were each compared to the simultaneously performed LGA measurements, and not to the initial measurement. However, when using the CPAs with intentions for diagnosis, a change over time might impact the result. This should be taken into account when performing repeated measurements.

## Conclusion

To conclude, all tested products appeared to be suitable to assess tremor in terms of frequency as they did not significantly differ from the gold standard with regard to peak frequency. The results of the amplitude at peak frequency differed between the LGA and the CPAs. Differences in variability of the measurements were observed, with less variability in resting arm than in extended arm position. For clinical purposes, it may be relevant to detect changes in tremor amplitude, as pharmacological intervention may lead to changes in amplitude rather than in frequency [5]. In order to precisely capture a change in the tremor amplitude, the variability of the amplitude measurement should be as low as possible. The optimal position to measure effects on tremor would be the resting arm position and a cross-over design is recommended to reduce the variability of the measurements.

## Supplementary information

**Additional file 1: Figure S1.** Limits of agreement of amplitude at peak frequency between gold standard and consumer products in all patients in resting arm position. **Figure S2.** Limits of agreement of amplitude at peak frequency between gold standard and consumer products in all patients in extended arm position. **Figure S3.** Limits of agreement of amplitude at peak frequency between gold standard and consumer products in Essential Tremor patients in resting arm position. **Figure S4.** Limits of agreement of amplitude at peak frequency between gold standard and consumer products in Essential Tremor patients in extended arm position. **Figure S5.** Limits of agreement of amplitude at peak frequency between gold standard and consumer products in Parkinson’s Disease patients in resting arm position. **Figure S6.** Limits of agreement of amplitude at peak frequency between gold standard and consumer products in Parkinson’s Disease patients in extended arm position. **Figure S7.** Mounting of standard LGA (Biometrics ACL300). **Figure S8.** Resting arm position. **Figure S9.** Extended arm position. **Figure S10.** Extended arm position with phone. **Figure S11.** Resting arm position with phone. **Table S1.** Minimum detectable change for each device for frequency and amplitude.

## Data Availability

The datasets used and/or analyzed during the current study are available from the corresponding author on reasonable request.

## Abbreviations

CCMO: Dutch Competent Authority
CPA: Consumer product accelerometers
ET: Essential Tremor
LGA: Laboratory-grade accelerometers
MW: MetaWear
PD: Parkinson’s disease
