# Supplementary material for: Quantification of tremor using consumer product accelerometry is feasible in patients with essential tremor and Parkinson’s disease: a comparative study
Source: J Clin Mov Disord. 2020 Apr 7;7:4. doi: 10.1186/s40734-020-00086-7 (PMC7137336; doi:10.1186/s40734-020-00086-7)
Supplement: Supplementary file 1 — Additional file 1: Figure S1. Limits of agreement of amplitude at peak frequency between gold standard and consumer products in all patients in resting arm position. Figure S2. Limits of agreement of amplitude at peak frequency between gold standard and consumer products in all patients in extended arm position. Figure S3. Limits of agreement of amplitude at peak frequency between gold standard and consumer products in Essential Tremor patients in resting arm position. Figure S4. Limits of agreement of amplitude at peak frequency between gold standard and consumer products in Essential Tremor patients in extended arm position. Figure S5. Limits of agreement of amplitude at peak frequency between gold standard and consumer products in Parkinson’s Disease patients in resting arm position. Figure S6. Limits of agreement of amplitude at peak frequency between gold standard and consumer products in Parkinson’s Disease patients in extended arm position. Figure S7. Mounting of standard LGA (Biometrics ACL300). Figure S8. Resting arm position. Figure S9. Extended arm position. Figure S10. Extended arm position with phone. Figure S11. Resting arm position with phone. Table S1. Minimum detectable change for each device for frequency and amplitude. [file 40734_2020_86_MOESM1_ESM.docx]

**Supplementary figures**

**
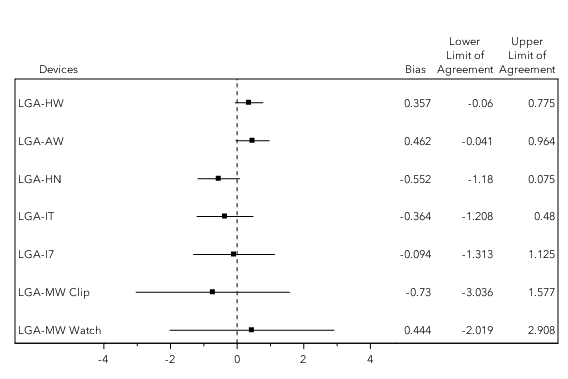
**

**Supplementary Figure 1.** Limits of agreement of amplitude at peak frequency between gold standard and consumer products in all patients in resting arm position. AW = Apple watch 2; HN = Huawei Nexus 6P; HW = Huawei watch; IT = iPod Touch 5; I7 = iPhone 7; MC = Meta Wear Clip; MW = Meta Wear watch.

**
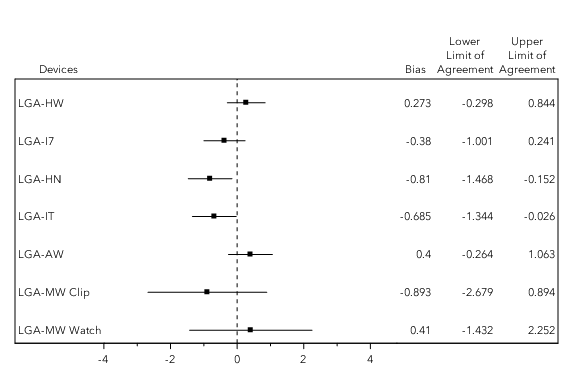
**

**Supplementary Figure 2.** Limits of agreement of amplitude at peak frequency between gold standard and consumer products in all patients in extended arm position. AW = Apple watch 2; HN = Huawei Nexus 6P; HW = Huawei watch; IT = iPod Touch 5; I7 = iPhone 7; MC = Meta Wear Clip; MW = Meta Wear watch.

**
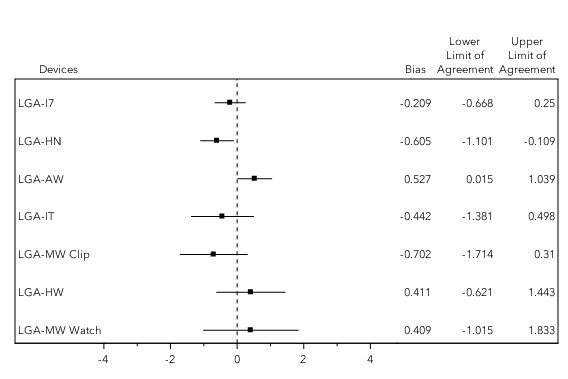
**

**Supplementary Figure 3.** Limits of agreement of amplitude at peak frequency between gold standard and consumer products in Essential Tremor patients in resting arm position. AW = Apple watch 2; HN = Huawei Nexus 6P; HW = Huawei watch; IT = iPod Touch 5; I7 = iPhone 7; MC = Meta Wear Clip; MW = Meta Wear watch.

**
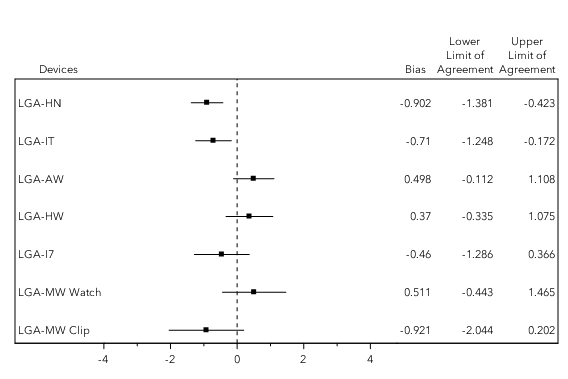
**

**Supplementary Figure 4.** Limits of agreement of amplitude at peak frequency between gold standard and consumer products in Essential Tremor patients in extended arm position. AW = Apple watch 2; HN = Huawei Nexus 6P; HW = Huawei watch; IT = iPod Touch 5; I7 = iPhone 7; MC = Meta Wear Clip; MW = Meta Wear watch.

**
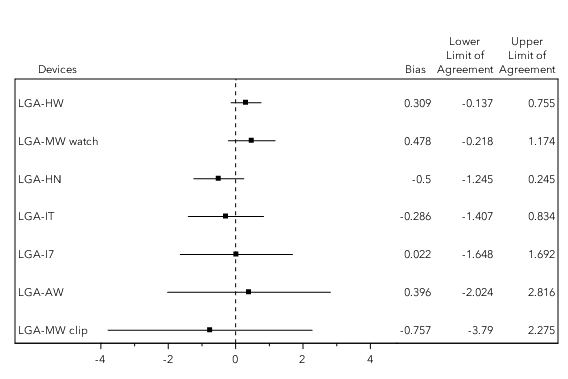
**

**Supplementary Figure 5.** Limits of agreement of amplitude at peak frequency between gold standard and consumer products in Parkinson’s Disease patients in resting arm position. AW = Apple watch 2; HN = Huawei Nexus 6P; HW = Huawei watch; IT = iPod Touch 5; I7 = iPhone 7; MC = Meta Wear Clip; MW = Meta Wear watch.


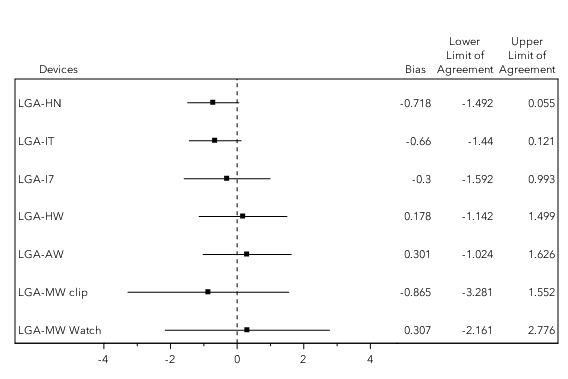


**Supplementary Figure 6.** Limits of agreement of amplitude at peak frequency between gold standard and consumer products in Parkinson’s Disease patients in extended arm position. AW = Apple watch 2; HN = Huawei Nexus 6P; HW = Huawei watch; IT = iPod Touch 5; I7 = iPhone 7; MC = Meta Wear Clip; MW = Meta Wear watch.


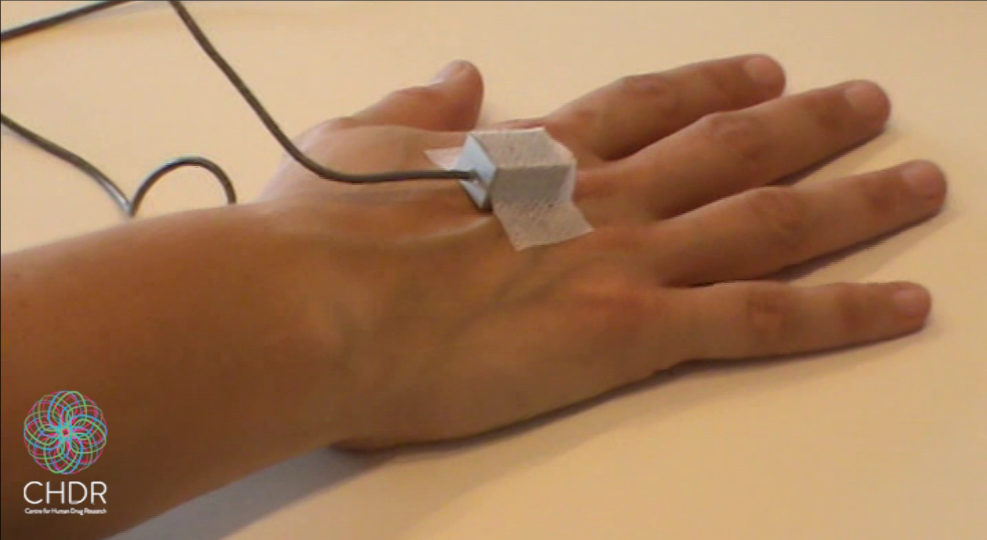


**
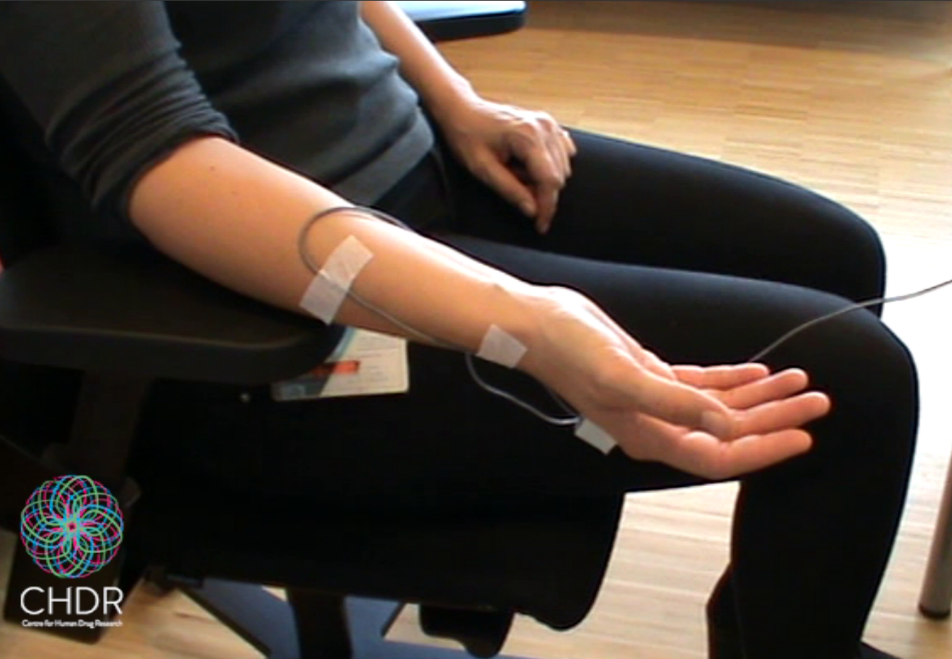
Supplementary figure 7.** Mounting of standard LGA (Biometrics ACL300)

**Supplementary figure 8.** Resting arm position

**
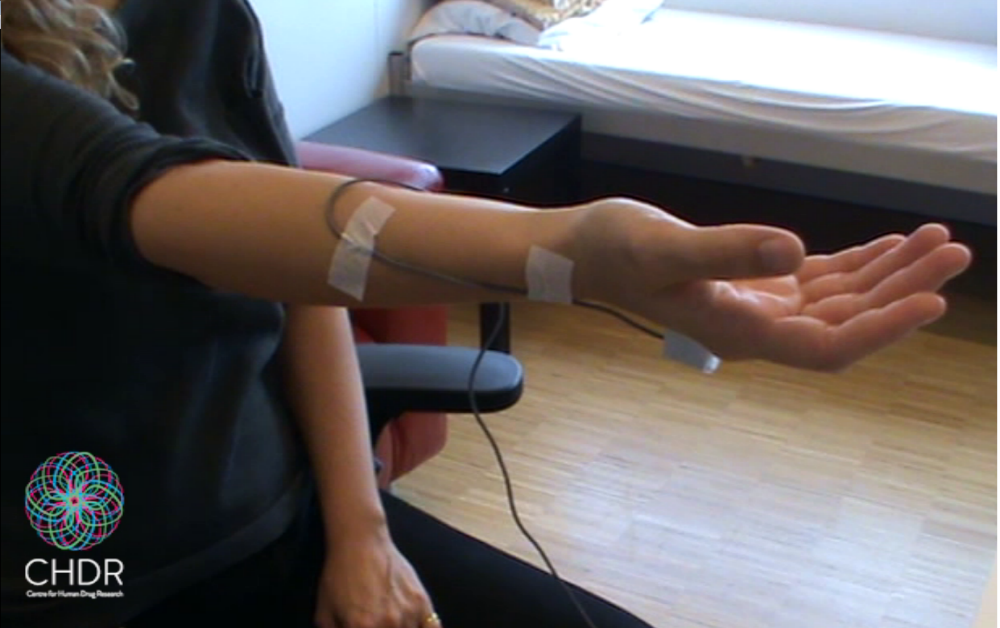

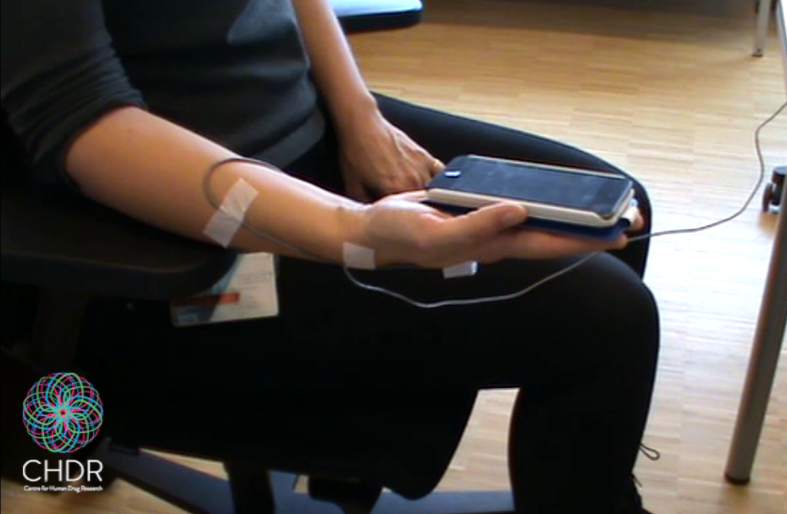

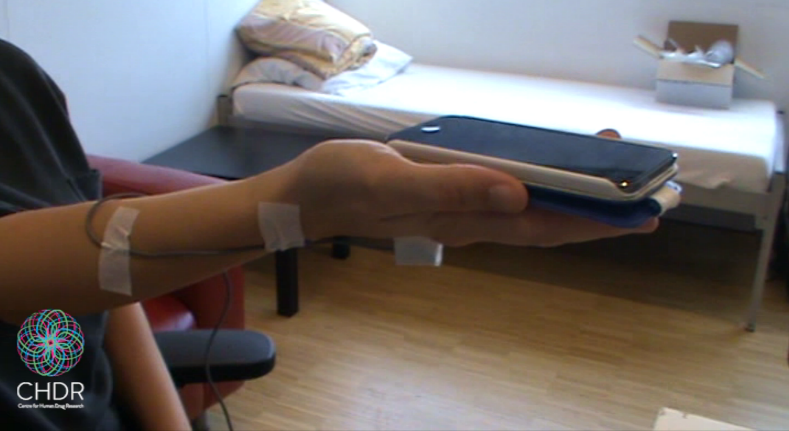
Supplementary figure 9.** Extended arm position

**Supplementary figure 10.** Extended arm position with phone

**Supplementary figure 11.** Resting arm position with phone

**Supplementary table 1.** Minimum detectable change for each device for frequency and amplitude

| **Device** | **Minimum detectable change Frequency (Hz)** | |
| --- | --- | --- |
| **Position** | **Resting arm** | **Extended arm** |
| Standard (Biometrics ACL300)  Baseline 1* | 0.654 | 1.229 |
| Standard (Biometrics ACL300)  Baseline 2** | 1.063 | 1.288 |
| iPhone 7 | 0.812 | 1.017 |
| iPod Touch 5 | 0.872 | 1.037 |
| Apple Watch 2 | 0.918 | 1.519 |
| Huawei Nexus 6P | 0.918 | 0.879 |
| Huawei watch | 1.024 | 1.367 |
| MW watch | 0.680 | 1.004 |
| MW clip | 0.859 | 0.793 |

*Baseline before the hand held devices

**Baseline before the wrist worn devices

| **Device** | **Minimum detectable change Amplitude ((mG)^2/Hz)** | |
| --- | --- | --- |
| **Position** | **Resting arm** | **Extended arm** |
| Standard (Biometrics ACL300)  Baseline 1* | 1538.40 | 81.64 |
| Standard (Biometrics ACL300)  Baseline 2** | 868.86 | 42.39 |
| iPhone 7 | 467.98 | 324.65 |
| iPod Touch 5 | 1456.18 | 2321.26 |
| Apple Watch 2 | 630.35 | 78.53 |
| Huawei Nexus 6P | 1692.40 | 1794.50 |
| Huawei watch | 863.38 | 192.04 |
| MW watch | 546.57 | 84.82 |
| MW clip | 10247.21 | 4383.24 |

*Baseline before the hand held devices

**Baseline before the wrist worn devices
